# GnRHa protects the ovarian reserve by reducing endoplasmic reticulum stress during cyclophosphamide-based chemotherapy

**DOI:** 10.1038/s41523-021-00340-7

**Published:** 2021-10-07

**Authors:** Xiaolin Li, Sixuan Liu, Xuan Chen, Run Huang, Lisi Ma, Huaiyu Weng, Yang Yu, Xiangyun Zong

**Affiliations:** 1 Department of Breast Surgery, Shanghai Jiao Tong University Affiliated Shanghai Sixth People’s Hospital, 600 Yishan Road, Shanghai, 200233 China; 2 Institute of Cancer and Basic Medicine, Cancer Hospital of the University of Chinese Academy of Science (Zhejiang Cancer Hospital), 1 East Banshan Road, Hangzhou, 310022 China

**Keywords:** Translational research, Autophagy

## Abstract

Chemotherapy-induced ovarian dysfunction is a serious adverse effect in premenopausal patients with cancer. Gonadotrophin-releasing hormone analogs (GnRHa) protect ovarian function, but its molecular mechanisms have not yet been determined. In this study, we attempted to determine the previously unknown molecular mechanism by which such protection occurs. Serum anti-Müllerian hormone (AMH) levels were tested in tumor-bearing nude mice, a series of exploratory experiments were conducted. We discovered that GnRHa protects granulosa cells from chemotherapeutic toxicity in vivo and in vitro. We also showed that CTX-induced endoplasmic reticulum stress inhibits the secretion of AMH, and treatment with GnRHa relieves ER stress and the subsequent unfolded-protein response by modulating mTOR signaling to induce autophagy. The results of mechanistic studies indicated that GnRHa-modulated mTOR signaling to induce autophagy, which alleviated CTX-induced ER stress and promoted the secretion of AMH.

## Introduction

Treatment for most patients with breast cancer involves chemotherapy, which usually has toxic adverse effects on the ovaries and destroys the ovarian reserve, leading to infertility and premature ovarian insufficiency (POI)^1–3^. Following chemotherapy, more than half of premenopausal patients with breast cancer experience amenorrhea, which is a widely used clinical marker of chemotherapy-induced POI. According to the latest guidelines, first-line chemotherapeutic regimens for breast cancer are based on cyclophosphamide (CTX), an alkylating agent that has a pro-apoptotic adverse effect on ovarian granulosa cells. Because these cells have important regulatory roles in the ovaries, CTX chemotherapy induces dose-dependent amenorrhea. Studies have shown the rate of amenorrhea with CTX range from 50% to 95%, and amenorrhea in older women is more likely to be irreversible^1,4–6^.

The ovarian reserve is an indication of the capacity of the ovary to provide oocytes that are capable of fertilization. Because levels of many markers vary naturally throughout the course of the menstrual cycle so that it is difficult to obtain a steady-state measurement that relates to the ovarian reserve. Serum markers, such as follicle-stimulating hormone, luteinizing hormone, inhibin B, and anti-Müllerian hormone (AMH), have been assessed for their potential in the evaluation of the ovarian reserve^7^. Among these markers, AMH (a protein secreted by the granulosa cells of preantral follicles in the ovary) is a relatively stable indicator of the ovarian reserve that does not vary significantly according to the position in the menstrual cycle, or from one cycle to the next. Therefore, in numerous studies, AMH has been adopted as a biomarker for ovarian reserve during the postchemotherapy follow-up period^8,9^.

An important consideration in the treatment of premenopausal patients with breast cancer is the desire to preserve ovarian function. Treatment with gonadotrophin-releasing hormone analog (GnRHa) is used as a strategy to avoid chemotherapy-induced POI. Evidence from randomized control trials (RCTs) indicates that GnRHa is associated with the preservation of the ovarian reserve when administered concurrently with chemotherapy^10^. The results of an RCT involving patients with hormone receptor-negative breast cancer also demonstrated a protective effect of GnRHa for ovarian function^11^. A recent prospective cohort study conducted by wang et al.^12^ further indicated that GnRHa had a protective effect in young breast cancer patients in terms of AMH and antral follicle count (AFC) during chemotherapy.

**Fig. 1:**
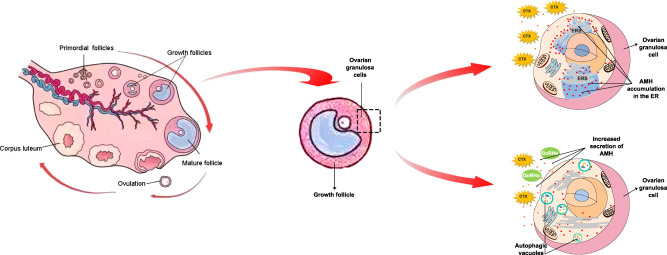
Graphical abstract of the mechanistic study. Cyclophosphamide induces AMH accumulation in the endoplasmic reticulum (ER), further inhibits the secretion of AMH by induction of ER stress in ovarian granulosa cells, leading to depletion of ovarian reserve. GnRHa co-treatment alleviates ER stress by inhibiting the mTOR pathway to induce autophagy, which would ultimately result in AMH secretion. Secreted AMH arrives at the ovaries and protects the ovarian reserve, attenuating chemotherapy-induced ovarian dysfunction and preventing POI.

The mechanism underlying the protection of ovarian function by GnRHa during chemotherapy, and the relationship between GnRHa treatment and serum AMH levels, have not yet been fully investigated. One possible mechanistic reason for the protective effect is that GnRHa restricts the blood supply to the ovary, thus reducing exposure to the toxic effects of chemotherapeutic agents^13^. A direct effect of GnRHa on ovarian function has been reported to be mediated through the GnRH receptor, but the exact molecular mechanism underlying this effect has not been investigated^14^. Another suggested mechanism for the reduction of chemotherapy-associated gonadotoxicity by GnRHa is the upregulation of an antiapoptotic molecule such as sphingosine-1-phosphate^15^. In this study, we attempted to determine the previously unknown molecular mechanism by which such protection occurs. Serum AMH levels were tested in tumor-bearing nude mice, in vivo and in vitro experiments including mechanistic studies were conducted (Fig. 1).

## Results

### GnRHa protects the ovarian reserve during chemotherapy in vivo

**Fig. 2:**
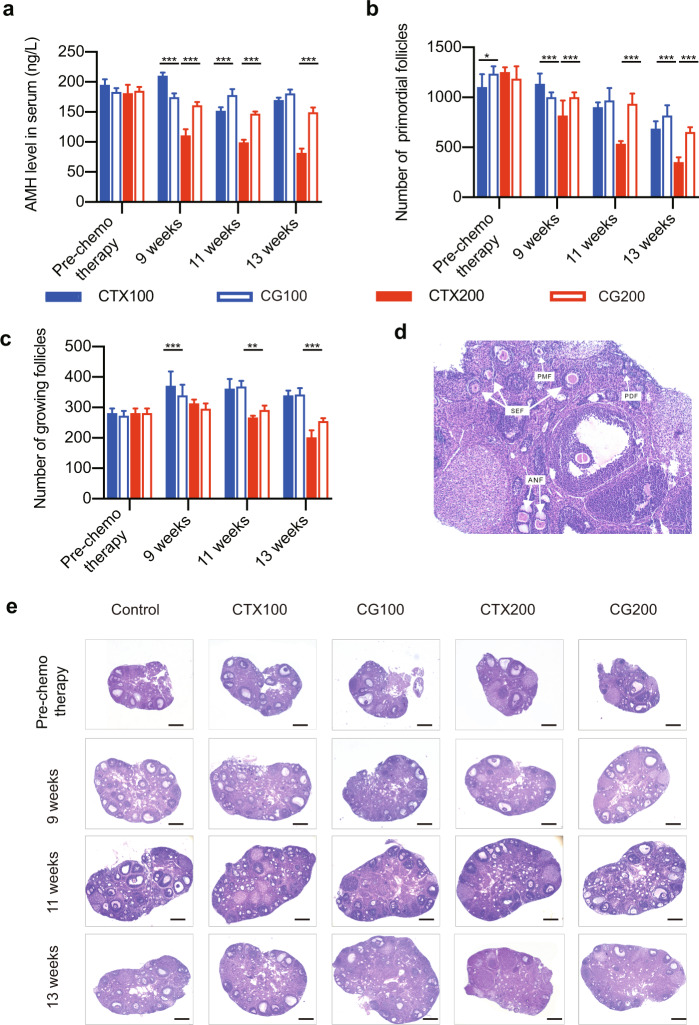
Serum AMH levels and ovarian-follicle counts in tumor-bearing nude mice. **a** Serum AMH levels at each time point after different interventions. **b** Number of primordial follicles per ovary in each group. **c** Number of growing follicles per ovary in each group. **d** H&E staining of normal ovarian section. **e** Histologic analysis of ovaries in each group after every sacrifice. CTX100, 100 mg/kg CTX. CG100, 100 mg/kg CTX+ Goserelin. CTX200, 200 mg/kg CTX. CG200, 200 mg/kg CTX+ Goserelin. Error bars show the standard error of the mean. Two-tailed Student’s *t* test was used for statistical analysis, *P < 0.05, **P < 0.001, and ***P < 0.0001.

To explore the effect of GnRHa on ovarian function, we constructed a tumor-bearing nude-mouse model by subcutaneously injecting mice with MCF7 cells. Tumor-bearing mice were treated with CTX at one of two doses, with or without four administrations of GnRHa, as illustrated in Supplementary Figure 1. To evaluate the ovarian reserve, blood samples were collected, and AMH was assessed by ELISA. In CTX-treated mice without GnRHa co-treatment, blood AMH levels declined as time progressed, demonstrating that CTX negatively affected ovarian reserve. Significant reductions in blood AMH levels occurred, especially at the higher CTX dose of 200 mg/kg (Fig. 2a). However, in mice co-treated with GnRHa and 100 mg/kg CTX, no reduction in AMH levels occurred with time. With co-treatment with GnRHa and 200 mg/kg CTX, a significant reduction in AMH levels occurred with time, but the reduction was much less than that observed in the absence of GnRHa (Fig. 2a).

Primordial and growing follicles were counted in ovaries from mice that were euthanized at different time points. The number of primordial follicles fell significantly (and dose-dependently) over time with CTX treatment (Fig. 2b and Fig. 2e). This reduction still occurred, but was not as pronounced, in mice with GnRHa co-treatment (Fig. 2b). By contrast, treatment with CTX did not result in a reduction in numbers of growing follicles (including primary follicles, secondary follicles, and antral follicles) (Fig. 2d and Fig. 2e). Indeed, with 100 mg/kg CTX, the numbers of growing follicles were significantly higher after 9 and 11 weeks of chemotherapy than pre-treatment, indicating that CTX activated the growth of the quiescent primordial follicle population (Fig. 2c). However, no increase in the numbers of growing follicles was seen when mice were treated with the higher CTX dose. Even at week 13, the number of growing follicles was significantly lower than pre-treatment (Fig. 2c). Co-treatment with GnRHa had little effect on the numbers of growing follicles, compared with CTX alone (Fig. 2c, e). These results suggested that GnRHa acts to relieve CTX-induced gonadotoxicity.

### CTX inhibits AMH secretion in vitro

**Fig. 3:**
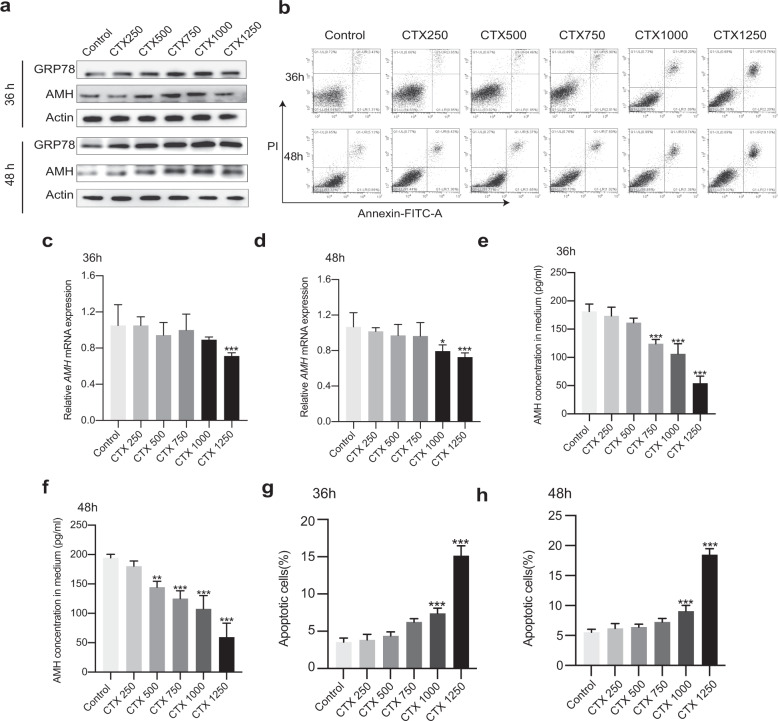
Cyclophosphamide inhibits AMH secretion in vitro. **a** Representative western blot analysis of GRP78 and AMH from KGN cells at 36 h and 48 h after injection of vehicle or CTX at doses of 250–1250 µg/mL. **b** Apoptosis assay of KGN cells 36 h and 48 h after injection of vehicle or CTX at doses of 250–1250 µg/mL. Compared with untreated control cells, the level of apoptosis was significantly higher with ≥750 µg/mL CTX. **c**, **d** *AMH mRNA* levels of KGN cells verified by qPCR. **e**, **f** The concentration of AMH in the cell-culture medium declined as the CTX dose increased. **g**, **h** One-way ANOVA analysis of apoptotic cells displayed on b. All error bars represent the standard error of the mean. *P < 0.05, **P < 0.001, and ***P < 0.0001.

We studied the effect of CTX on granulosa cells, which play important roles in ovarian follicular development and are the main target of CTX. To this end, we exposed human ovarian granulosa-like tumor (KGN) cells to CTX and measured AMH expression. Western blotting results demonstrated that levels of AMH protein were higher in extracts of KGN cells treated with CTX than in extracts of untreated controls, with AMH levels seeming to increase dose-dependently with the dose of CTX (Fig. 3a). However, *AMH* mRNA levels did not change significantly in the presence of <1000 µg/mL CTX, whereas at 1250 µg/mL CTX (and at 1000 µg/mL CTX after 48 h), the *AMH* mRNA level was significantly lower than in untreated control cells (Fig. 3c, d). In addition, the percentage of apoptotic cells increased with the CTX dose, and at ≥1000 µg/mL CTX (after 36 h) it was significantly greater than the level in the untreated controls (Fig. 3b and Fig. 3g, h). Notably, the concentration of AMH in the cell-culture medium declined as the CTX dose increased, and at ≥750 µg/mL CTX (after 36 h) it was significantly lower than the concentration in the medium of untreated cells, indicating that CTX suppressed the secretion of AMH into the medium (Fig. 3e, f). The patterns of reduction of secreted AMH at 36 h and 48 h seemed to be the inverse of the patterns of increasing AMH protein levels in the cell lysates. Overall, these results suggested that CTX-inhibited the secretion of AMH from the granulosa cells before they went into apoptosis.

### GnRHa reverses CTX-induced inhibition of AMH secretion

**Fig. 4:**
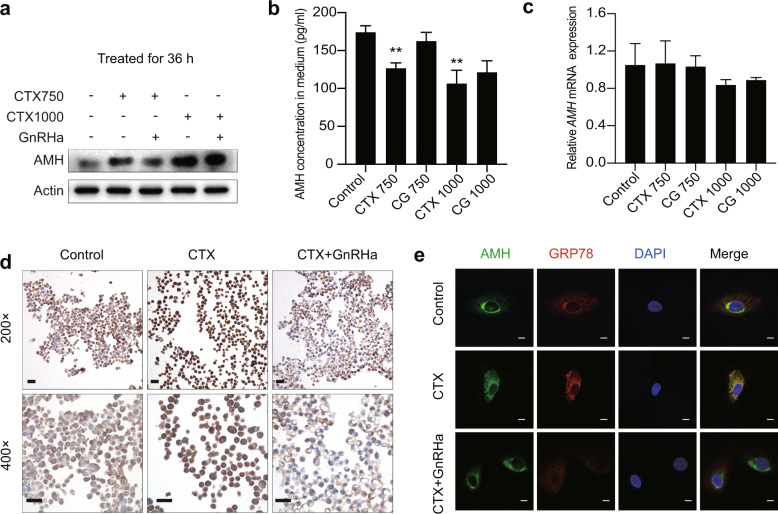
GnRHa reverses the CTX-induced inhibition of AMH secretion. KGN cells were treated with CTX or CTX plus GnRHa for 36 h. **a** AMH concentration in KGN cells verified by western blot. Compared with CTX-treated cells, the accumulation of AMH was remitted by co-treatment with GnRHa. **b** AMH level in cell-culture medium verified by ELISA. Significant reductions of secreted AMH were prevented by co-treatment with GnRHa. **c** *AMH mRNA* levels of cells verified by qPCR. **d** Typical immunocytochemistry images demonstrate the accumulation of AMH in the cytoplasm of cells treated with 750 µg/mL CTX, which was released by co-treatment with GnRHa. **e** Colocalization of AMH and GRP78 in KGN cells. Cells were transfected with a plasmid expressing FLAG-tagged AMH, and were treated with the indicated reagents for 36 h. Yellow in the merged images indicates the colocalization of AMH and GRP78. Nuclei were stained with DAPI (4’,6’-diamidino-2-phenylindole). CTX750, 750 µg/mL CTX. CG750, 750 µg/mL CTX+ Goserelin. CTX1000, 1000 µg/mL CTX. CG1000, 1000 µg/mL CTX + Goserelin. Scale bars represent 100 µm. Error bars show the standard error of the mean. One-way ANOVA was used for **b**–**c**, *P < 0.05, **P < 0.001, and ***P < 0.0001.

Next, to further characterize the CTX-induced inhibition of AMH secretion and the effect of GnRHa co-treatment, we treated granulosa cells with combinations of GnRHa and CTX. The level of AMH protein in the lysate of cells treated with GnRHa and 750 µg/mL CTX was lower than that in cells treated only with 750 µg/mL CTX. With 1000 µg/mL CTX, no obvious difference was observed between cells with and without GnRHa co-treatment (Fig. 4a). GnRHa treatment did not affect *AMH* mRNA expression in the presence of either 750 µg/mL or 1000 µg/mL CTX (Fig. 4c). Consistent with the immunoblotting findings, GnRHa co-treatment prevented the CTX-induced reduction in the secretion of AMH, so that the AMH concentrations in the media of cells treated with 750 µg/mL or 1000 µg/mL CTX, in the presence of GnRHa, were not significantly different from those for untreated control cells (Fig. 4b). To further characterize the effect of CTX and/or GnRHa on AMH secretion, immunocytochemistry and immunofluorescence were performed. For each experimental group (untreated control, 750 µg/mL CTX, and 750 µg/mL CTX+ GnRHa), cells from three treatments were embedded in a single paraffin block to reduce any bias resulting from experimental variation. Immunocytochemistry showed that, compared with untreated control cells, high levels of AMH accumulation occurred in the cytoplasm of KGN cells treated with 750 µg/mL CTX. Compared with the CTX-treated cells, staining for AMH was less intense in cells co-treated with CTX and GnRHa (Fig. 4d). Following transfection of a vector directing expression of FLAG-tagged AMH into KGN cells, results from immunofluorescence microscopy suggested a higher level of colocalization of AMH and the ER marker GRP78 in cells treated with CTX than in untreated control cells (Fig. 4e). Little colocalization of AMH and GRP78 was observed in cells co-treated with CTX and GnRHa. Overall, our results indicated that CTX caused inhibition of AMH secretion by inducing AMH accumulation in the ER and that these effects were reversed by GnRHa co-treatment.

### GnRHa counteracts CTX-induced ER stress

**Fig. 5:**
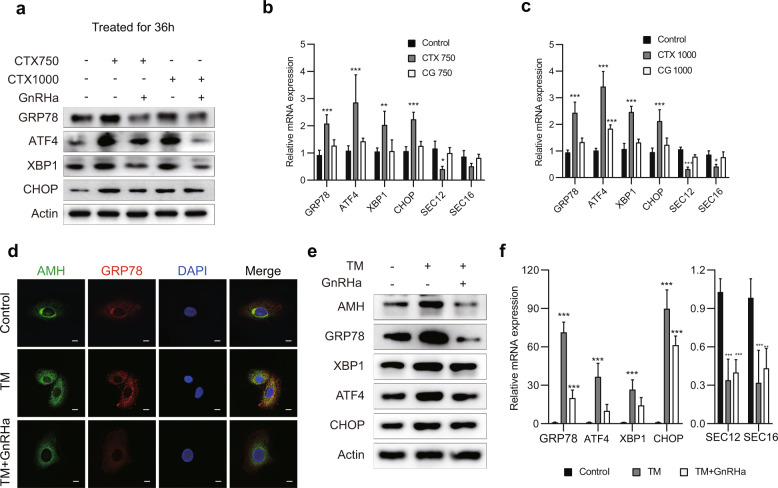
GnRHa counteracts CTX-induced ER stress. **a** Expression of proteins including GRP78, ATF4, XBP1, and CHOP in the UPR pathway was assessed by western blotting. KGN cells were treated with 750 or 1000 µg/mL CTX, with or without GnRHa, for 36 h. **b**, **c** KGN cells were treated with CTX or CTX plus GnRHa for 36 h. mRNA levels were assessed by RT-qPCR for genes encoding proteins involved in the UPR (*GRP78*, *XBP1*, *ATF4,* and *CHOP*), and for genes encoding secretion-related proteins (*SEC12* and *SEC16*). Those mRNAs were upregulated by CTX and downregulated when co-treated with GnRHa. **d** Typical immunofluorescence staining indicated the localization of AMH within KGN cells. Yellow in the merged images indicates the colocalization of AMH and GRP78. Nuclei were stained with DAPI. Scale bars represent 100 µm. **e** TM was used for simulation of ER stress, and expressions of proteins in the UPR pathway were assessed by western blotting, the results were consistent with the CTX-treated cells. **f** TM-induced upregulation of UPR sensors was confirmed by RT-PCR for mRNA expression. One-way ANOVA was used for (**b**, **c**, and **f**), error bars show the standard error of the mean. *P < 0.05, **P < 0.001, and ***P < 0.0001.

Protein accumulation in the ER triggers activation of the ER stress pathway UPR^16^. In fact, western blotting results with extracts of KGN cells suggested that CTX promoted the expression of the chaperone molecule GRP78, which is the upstream regulator of UPR sensors (Fig. 3a). Therefore, we investigated UPR activation in CTX- and/or GnRHa-treated KGN cells by measuring the expression of factors downstream from GRP78. Western blotting showed that ATF4, XBP1, and CHOP were obviously upregulated in the presence of CTX (Fig. 5a). Meanwhile, RT-qPCR demonstrated the significant upregulation of *GRP78*, *ATF4*, *XBP1*, and *CHOP* mRNA expression (compared with levels in untreated control cells), suggesting that CTX-induced ER stress (Fig. 5b, c). Co-treatment with GnRHa prevented the CTX-induced upregulation of these UPR sensors, suggesting an important role for GnRHa in blocking CTX-induced ER stress (Fig. 5a). This role was further investigated by the use of TM to induce ER stress and the UPR in KGN cells. TM is an inducer of endoplasmic reticulum stress (ERS). TM-induced upregulation of UPR sensors, which was reduced by GnRHa co-treatment, was suggested by western blotting for protein expression, and confirmed by RT-PCR for mRNA expression (Fig. 5e, f). Western blotting also suggested TM-induced upregulation of AMH protein levels in KGN cell lysates, which was prevented by GnRHa co-treatment (Fig. 5e). Similarly, immunofluorescence microscopy demonstrated high levels of colocalization of AMH and GRP78 in the ER in cells treated with TM, but not in cells co-treated with TM and GnRHa (Fig. 5d).

Because our results indicated downregulation of AMH secretion by CTX, we investigated the effects of ER stress on the expression of the protein-secretion-related proteins SEC12 and SEC16. Compared with the levels in untreated control cells, mRNA expression of *SEC12* and *SEC16* was significantly downregulated by treatment with CTX or TM (Fig. 5b, c and Fig. 5f). Downregulation by CTX was effectively prevented by GnRHa co-treatment (Fig. 5b, c), whereas downregulation by TM was only slightly affected by GnRHa (Fig. 5f).

**Fig. 6:**
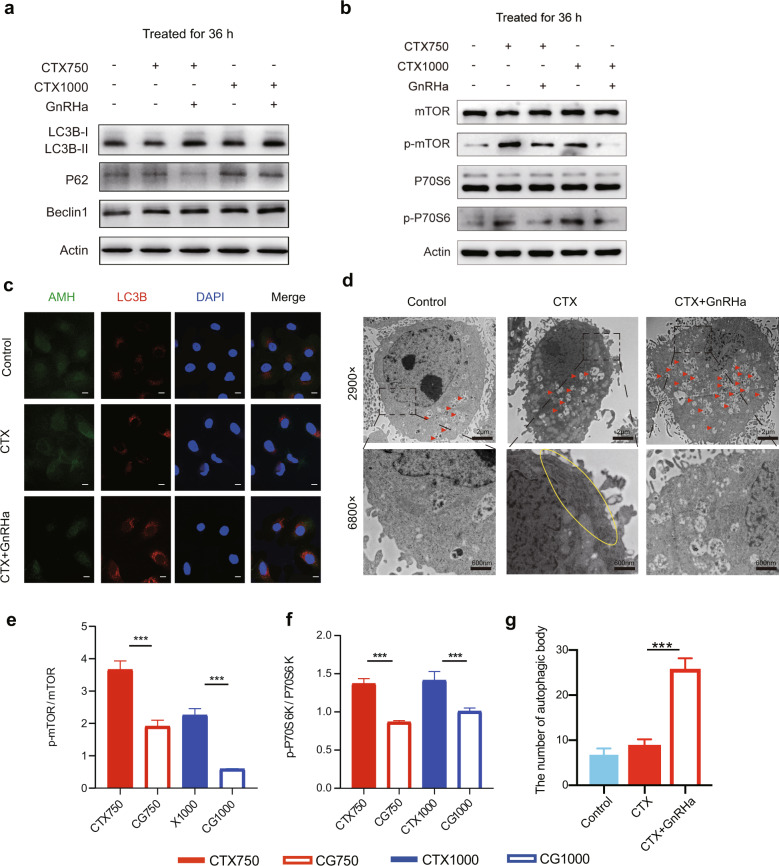
GnRHa induces autophagy to relieve ER stress by inhibiting mTOR signaling. **a** Expression of proteins in autophagy was assessed by western blotting. Compared with CTX-treated cells, LC3B-II and Beclin-1 were higher, P62 were lower in cells co-treated with CTX and GnRHa. **b** Expressions of proteins in mTOR pathway were assessed by western blotting. **c** Typical immunofluorescence staining, indicating the localization of AMH and LC3B within KGN cells. Yellow in the merged images indicates the colocalization of AMH and LC3B. Nuclei were stained with DAPI. Scale bars represent 100 μm. **d**, **g** Representative transmission electron microscopy results are shown for KGN cells treated with 750 µg/mL CTX, with or without GnRHa, for 36 h. Red arrowheads represent autophagic vacuoles; the yellow oval marks an area of ER stress. When the cells were co-treated with CTX and GnRHa, the numbers of autophagosomes appeared to be considerably higher. **e**, **f** Co-treatment with CTX and GnRHa reduced mTOR and P70S6K phosphorylation compared with CTX only. Compared with untreated control cells, the phosphorylation of mTOR and P70S6K were higher in the CTX-treated KGN cells. Data are expressed as mean ± SD of three independent experiments. Error bars show the standard error of the mean. Two-tailed Student’s *t* test was used for (**e**, **f**), two-way ANOVA (Tukey’s test) was used for **g**, *P < 0.05, **P < 0.001, and ***P < 0.0001.

To further study the mechanism underlying the regulation of AMH secretion by CTX and GnRHa, we assessed the micromorphology of organelles in KGN cells. Compared with untreated control cells, TEM showed an increase and dilation of ER in KGN cells treated with 750 µg/mL CTX for 36 h, which was not seen when the cells were co-treated with CTX and GnRHa (Fig. 6d).

Overall, these results demonstrated that GnRHa alleviated CTX-induced ER stress and UPR, and thus promoted AMH secretion.

### GnRHa-induced autophagy relieves ER stress

To further understand how GnRHa prevented CTX-induced ER stress, we hypothesized that autophagy was involved in the GnRHa-mediated modulation of CTX-induced ER stress. In KGN cells treated with CTX and in untreated control cells, similar numbers of autophagosomes were identified by TEM (Fig. 6d, g). However, when the cells were co-treated with CTX and GnRHa, the numbers of autophagosomes appeared to be considerably higher (Fig. 6d and Fig. 6g). Levels of the autophagy markers LC3B, P62, and beclin-1 in KGN cells were assessed by western blotting (Fig. 6a). In cells co-treated with CTX and GnRHa, the results seemed to show enhancement of conversion of the soluble form of LC3B (LC3B-I) to the insoluble form (LC3B-II, by covalent conjugation with phosphatidylethanolamine), which is a hallmark of autophagy^17^. Beclin-1 is one of the main regulators of autophagy and has a central role in autophagosome formation and maturation^18^, and its expression is higher in cells co-treated with CTX and GnRHa than in untreated cells or cells treated with CTX alone (Fig. 6a). Furthermore, the abundance of P62, an LC3B-binding protein, and receptor that is degraded during the autophagic process^19^, was lower in cells co-treated with CTX and GnRHa than in cells treated with CTX alone (Fig. 6a). Immunofluorescence microscopy demonstrated low levels of LC3B expression and colocalization with AMH when KGN cells were treated with CTX alone, and higher levels when cells were co-treated with CTX and GnRHa (Fig. 6c). Overall, these results suggested that GnRHa-induced autophagy relieved CTX-induced ER stress.

### GnRHa inhibits mTOR pathway to induce autophagy

To further explore the specific molecular mechanism of GnRHa-induced autophagy, we evaluated the effect of CTX and/or GnRHa treatment for 36 h on the mTOR signaling pathway in KGN cells. The kinase mammalian/mechanistic target of rapamycin is an autophagy-suppressive regulator and inhibition of mTOR leads to induction of autophagy^20^. Western blotting showed that CTX increased phosphorylation of mTOR and phosphorylated P70S6K, whereas co-treatment with GnRHa decreased the expression of phosphorylated mTOR and phosphorylated p70S6K (Fig. 6b and Fig. 6e, f). These results demonstrated that the CTX treatment led to the hyperactivation of the mTOR signaling pathway, and co-treatment with GnRHa can inhibit mTOR signaling pathway, which induces increased autophagy to relieve CTX-induced ER stress.

### GnRHa relieves ER stress by inhibiting mTOR pathway to induce autophagy in CTX-treated mice

**Fig. 7:**
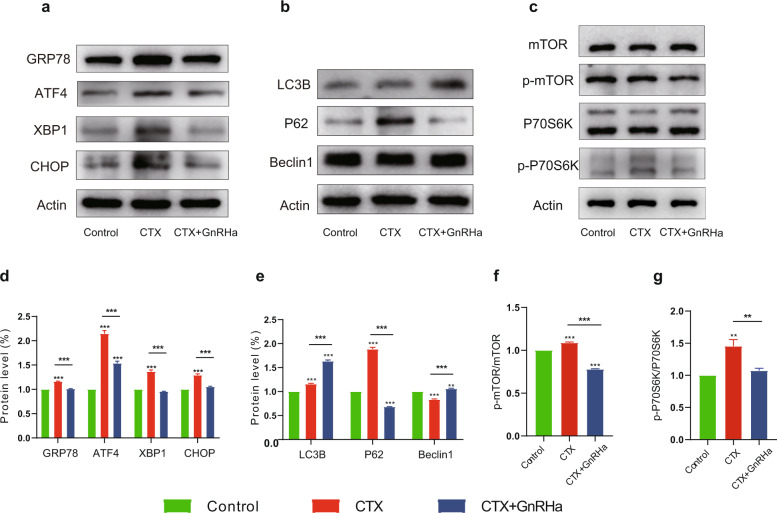
GnRHa relieves ER stress by inhibiting mTOR pathway to induce autophagy in CTX-treated mice. To explore the effect of CTX and/or GnRHa on mouse ovaries, 8-week-old tumor-bearing nude mice were treated with saline, 200 mg/kg CTX, and 200 mg/kg CTX + GnRHa, and ovarian protein were extracted 1 week after treatment. **a** Expression of proteins in the ER stress pathway was assessed by western blotting. **b** Expressions of proteins in the autophagy were assessed by western blotting. **c** Expressions of proteins in the mTOR pathway were assessed by western blotting. **d** The GRP78, ATF4, XBP1, and CHOP increased significantly in CTX-treated ovaries than the control or CTX + GnRHa group. **e** Compared with CTX-treated mice, LC3B and Beclin-1 were increased, and P62 was decreased in coadministration with GnRHa mice. **f**, **g** The phosphorylation of mTOR and P70S6K were significantly increased in ovaries of CTX alone treated mice. Data are expressed as mean ± SD of three independent experiments. All error bars represent the standard error of the mean. Two-tailed Student’s *t* test was used for statistical analysis, *P < 0.05, **P < 0.001, and ***P < 0.0001.

To better understand the role of GnRHa in mouse ovaries, tumor-bearing nude mice were treated with saline, 200 mg/kg CTX, and 200 mg/kg CTX + GnRHa, and ovaries were removed 1 week after treatment. Total protein of mouse ovaries was extracted and was assessed by western blotting. The expression of UPR sensors GRP78, ATF4, XBP1, and CHOP increased significantly in CTX-treated ovaries than the control or CTX + GnRHa group (Fig. 7a and Fig. 7d). Coadministration with GnRHa significantly alleviated the CTX-induced increase expression of GRP78, ATF4, XBP1, and CHOP (Fig. 7a and Fig. 7d). The decreased expression of LC3B and Beclin-1 and increased expression of P62 in the CTX-treated ovaries indicated that CTX-inhibited autophagy (Fig. 7b and Fig. 7e). In the CTX + GnRHa group, LC3B and Beclin-1 were increased in association with decreased p62 (Fig. 7b and Fig. 7e). Furthermore, analysis of the mTOR signaling pathway showed that the phosphorylation of mTOR and P70S6K were significantly increased in ovaries of CTX alone treated mice and the mTOR signaling pathway was inhibited after GnRHa co-treatment (Fig. 7c and Fig. 7f, g).

Taken together, these results suggest that GnRHa inhibits mTOR signaling and induces autophagy to counteract CTX-induced ER stress which exerts a protective effect on the ovarian reserve.

## Discussion

The preservation of ovarian function during chemotherapy has been the subject of research for many years. The key to function preservation lies in answering the following questions: which chemotherapeutic agents are likely to cause ovarian damage in particular populations of patients; are there any indicators that can reflect ovarian function accurately^21^; what are the pathways through which chemotherapy drugs damage ovarian function; and what are the mechanisms by which protective agents defend the ovaries from damage, or reverse ovarian injury^22^? In this study, we investigated the mechanism underlying the protective effect of GnRHa against the gonadotoxicity caused by CTX and we found that CTX caused inhibition of AMH secretion by inducing AMH accumulation in the ER and that these effects were reversed by GnRHa co-treatment (Fig. 1). In the mouse model, CTX treatment decreased serum AMH levels and numbers of primordial follicles; co-treatment with GnRHa reduced the CTX-induced effects. In vitro, CTX promoted the accumulation of AMH in the ER and reduced the secretion of AMH. The results of mechanistic studies indicated that GnRHa inhibited the mTOR signaling pathway to induce autophagy, which alleviated CTX-induced ER stress and promoted the secretion of AMH.

Results from many clinical studies have shown that administration of GnRHa with chemotherapy is sufficient to protect against ovarian failure among women with breast cancer^10,11,23^. Lambertini et al.^24^ conducted a systematic review and meta-analysis of individual patient-level data, which provided favorable evidence for the efficacy and safety of GnRHa to reduce chemotherapy-induced POI and potentially improve future fertility in premenopausal patients with early breast cancer. However, in some studies, the results did not support the application of GnRHa, as there was insufficient evidence to assess outcomes related to GnRHa and ovarian function^25^. In the preclinical evidence of mouse experiments, although some experiments showed that GnRHa had no ovarian protection against CTX, the majority of these studies showed a positive effect for GnRHa treatment in preventing CTX-induced POI^2^. Our mouse experiment results showed that GnRHa counteracted the CTX-induced reduction in serum AMH in the mouse model. These results indicate that GnRHa has a protective effect on ovarian reserve.

Up to date, there are two main hypotheses about the mechanisms of GnRHa protecting ovarian function, which involves direct and indirect effects on the ovaries^2^. Scaruffi et al.^26^ treated cumulus cell-oocyte complexes with CTX and/or GnRHa, their data suggested that GnRHa had an antiapoptotic effect on cumulus cells instead of oocytes. We cultured KGN cells derived from ovarian follicular cells and determined the intracellular and extracellular levels of AMH. The addition of CTX to the medium increased AMH protein levels inside the cells, but decreased AMH secretion into the culture medium; co-treatment with GnRHa inhibited these effects. AMH mRNA expression did not change when KGN cells were treated with <1000 µg/mL CTX, but decreased at higher CTX doses, possibly as a result of the level of apoptosis at these higher CTX doses. In cells treated with CTX, AMH accumulated in the ER. Co-treatment with GnRHa promoted the secretion of AMH. Therefore, we propose that CTX inhibits the secretion of the AMH protein synthesized by granulosa cells and that GnRHa reverses this inhibitory effect.

We identified substantial accumulation of AMH in the ER of CTX-treated KGN cells, with lower levels in cells co-treated with CTX and GnRHa. It is possible that the accumulated AMH included unfolded or misfolded proteins, resulting in ER stress. We observed that GRP78 and downstream factors including ATF4, XBP1, and CHOP were upregulated in the presence of CTX, suggesting that CTX-induced ER stress and UPR. Co-treatment with GnRHa prevented the CTX-induced upregulation of these UPR sensors. Chemotherapy is an additional source of cellular stress for both cancer cells and normal somatic cells^27^. The UPR is favorable to cell survival under stressful conditions but leads to cell death in response to overwhelming ER stress. Here, we showed, in CTX-treated KGN cells, that GnRHa-mediated alleviation of ER stress increased AMH secretion. We suggest that, in the absence of ER stress, AMH protein can be correctly processed and secreted into the serum, further protecting primordial follicles from activation.

Autophagy is an important protective mechanism during ER stress^28,29^. We found that greater numbers of autophagosomes in cells treated with CTX and GnRHa than in cells treated with CTX only, suggesting that autophagy was involved in the GnRHa-mediated modulation of CTX-induced ER stress. This hypothesis was supported by western blotting results that suggested the promotion of autophagy by co-treatment with CTX and GnRHa. Although previous results have shown that ER stress induces autophagy^30^, we did not observe CTX-mediated upregulation of autophagy. However, autophagy has also been shown to alleviate inflammation, apoptosis, and ER stress^31^, and our results suggest that autophagy induced by GnRHa inhibits CTX-induced ER stress. Recent studies suggest autophagy is also a key biological process to preserve primordial follicles and impaired autophagy is the pathophysiological mechanism of POI^32,33^. Therefore, we have reason to hypothesize that GnRHa may protect the ovarian reserve function by inducing autophagy.

mTOR is a serine/threonine kinase that regulates cell growth, proliferation, and metabolism. At the same time, mTOR is a pharmacologic target for autophagy, which plays a crucial role in regulating autophagy^34^. The study of Kara et al.^35^ showed that mTOR inhibition preserved the ovarian reserve, primordial follicle counts, serum anti-Mullerian hormone levels, and fertility during genotoxic chemotherapy. Interestingly, the mTOR signaling pathway is also the main mechanism that regulates the quiescence and activation of primordial follicles: mTORC1 signal activates the downstream P70S6K/4EBP1-ribosomal protein S6 (rpS6) signal that promotes protein translation and ribosome biogenesis in oocytes, initiates follicle recruitment, and activates the growth and development of primordial follicles^36^. A large number of previous studies have shown that CTX activates the PI3K/PTEN-AKT-mTOR signaling pathway in ovaries^37,38^. Our results demonstrated that GnRHa co-treatment relieved CTX-induced hyperactivation of the mTOR signaling pathway in vivo and in vitro.

In conclusion, we make the proposition that the chemotherapeutic reagent CTX induces AMH accumulation in the ER, further inhibits the secretion of AMH by induction of ER stress in ovarian granulosa cells. GnRHa co-treatment alleviates ER stress by inhibiting mTOR pathway to induce autophagy, which would ultimately result in AMH secretion. Secreted AMH arrives at the ovaries and protects the ovarian reserve, attenuating chemotherapy-induced ovarian dysfunction and preventing POI, which is of clinical importance in the management of susceptible patients treated with chemotherapy. Our study found a new mechanism by which GnRHa protects ovarian function under CTX exposure, thus providing new ideas for ovarian function protection strategies. mTOR inhibitors, autophagy drivers, ER stress inhibitors, and AMH analogs as potential ovarian protective agents are all worthy of further study.

## Methods

### Animals

240 Balb/c-nu mice (6 weeks old, 18–20 g, female, obtained from Shanghai SLAC Laboratory Animal Co., Shanghai, China) were housed in sterile filter-capped cages. Each mouse was injected subcutaneously in the left flank with 1 × 10^7^ MCF7 cells in 100 μl of phosphate-buffered saline (PBS) and randomly assigned into one of five groups (Control, CTX100, CG100, CTX200, CG200, *n* = 48 in each group). Tumor growth was observed and recorded every 3 days. At 8 weeks old, mice were treated with a single intraperitoneal dose of 0.1 ml saline (Control group), 100 mg/kg CTX (CTX100 and CG100 groups), or 200 mg/kg CTX (CTX200 and CG200 groups). In addition, mice in CG100 and CG200 groups were treated with 90 µg/kg goserelin (AstraZeneca, Macclesfield, UK) 4 days before chemotherapy and every 4 days after chemotherapy. Twelve mice were randomly selected from each group to undergo heart-blood collection and ovary excision at the initiation of chemotherapy, 1 week after chemotherapy, and 3, and 5 weeks after ovarian function suppression (OFS) through administration of GnRHa (Supplementary Figure 1). The ovaries of 6 out of 12 mice killed in each group were removed for Western blot analysis. The remaining six ovaries of each group were fixed in formalin, embedded in paraffin, and stained with hematoxylin and eosin (H&E). The serum of all mice was isolated from blood samples for hormone measurements. All animal procedures were approved by the Animal Ethics Committee at Shanghai Jiao Tong University Affiliated Shanghai Sixth People’s Hospital and were carried out in accordance with the ‘Australian Code of Practice for the Care and Use of Animals for Scientific Purposes’.

### H&E staining

Ovaries obtained from euthanized mice were fixed in 4% paraformaldehyde overnight at 4 °C, then embedded in paraffin blocks. Each ovary was cut into 5-μm sections, and serial sections were mounted on glass slides and deparaffinized. Sections were stained with H&E following routine protocols after rehydration^39^. Light microscopy was used for visualization and image capture to determine follicle counts at different stages.

### Follicle counting

Follicle counting was performed as previously described^39^. In brief, the total number of follicles in each ovary was estimated by counting the follicles in every fifth section of H&E-stained whole ovaries and applying a fivefold correction factor. Follicles were classified into five stages: primordial, primary, secondary, antral, and follicles with a corpus luteum (Fig. 2d). Only follicles that had an oocyte nucleus were scored. In primordial follicles, the oocyte was surrounded by a layer of squamous granulosa cells. Primary follicles had a single layer of cuboidal granulosa cells. Secondary follicles had multiple layers of cuboidal granulosa cells. In antral follicles, an antrum was present in the granulosa cell layers. If an aberrant oocyte and multiple layers of pyknotic granulosa cells were observed, the follicle was classified as an atretic Primary follicle, secondary follicles, and antral follicles are collectively referred to as growing follicles.

### Cells and reagents

The KGN steroidogenic human ovarian granulosa-like tumor cell line was obtained from Guandao Bioengineering (Shanghai, China). The MCF7 human breast cancer cell line was obtained from the American Type Culture Collection. KGN and MCF7 cells were cultured in Dulbecco’s modified Eagle’s medium (Biopenony, Beijing, China) with 20% fetal bovine serum. To prevent bacterial contamination, penicillin and streptomycin were added to the medium at final concentrations of 100 U/mL and 100 mg/mL, respectively. Cells were incubated at 37 °C in a humidified incubator with air containing 5% CO_2_. CTX was obtained from Baxter Oncology (Halle Westfalen, Germany). GnRHa was obtained from AstraZeneca. Tunicamycin (TM, T8480) was obtained from Solarbio (Beijing, China). KGN cells were treated with CTX (250 µg/mL, 500 µg/mL, 750 µg/mL, 1000 µg/mL, or 1250 µg/mL), CTX (750 µg/mL) + GnRHa (100 µg/mL), CTX (1000 µg/mL) + GnRHa (100 µg/mL), TM (10 µg/mL), or TM (10 µg/mL) + GnRHa (100 µg/mL) for 36 h or 48 h.

### Plasmids

The FLAG-tagged AMH expression plasmid pENTER-AMH was purchased from Vigene Bioscience (Jinan, China), and the successful transfection of AMH was confirmed by nucleotide sequencing.

### Serum and medium AMH detection

Mouse blood (1 mL per animal) was collected by cardiac puncture. Blood was left to rest overnight at 4 °C. Serum was taken from the supernatant and stored at −20 °C until analysis. AMH levels in serum were detected with a Mouse AMH ELISA kit (XLPM0168, Biofine).

For AMH detection in medium, KGN cells were treated with CTX (250 µg/mL, 500 µg/mL, 750 µg/mL, 1000 µg/mL, or 1250 µg/mL), CTX (750 µg/mL) + GnRHa (100 µg/mL), TM (10 µg/mL), or TM (10 µg/mL) + GnRHa (100 µg/mL) for 36 h or 48 h, and DMEM medium was collected for AMH detection with a human AMH ELISA kit (11351, Biofine).

The ELISA to measure AMH was performed according to the manufacturer’s instructions. In brief, a 96-well plate was coated with mouse or human AMH antibody overnight at 4 °C and was blocked with 1% BSA/PBS and Tween 20 for 2 h at room temperature. The AMH standard and samples were added to the wells and incubated for 1 h at 37 °C. The rabbit polyclonal anti-AMH antibody was then added and incubated for 1 h at room temperature, and then donkey anti-rabbit IgG antibody conjugated to horseradish peroxidase (HRP) was added and incubated for 1 h at 4 °C. The plate was developed using 3,3′,5,5′-Tetra-methylbenzidine (TMB), and the light absorbance was read at 450 nm in a microplate reader (Vector2 1420; Perkin-Elmer).

### Immunocytochemistry

Immunocytochemistry was performed according to routine immunohistochemistry methodology. In brief, KGN cells fixed in PFA were paraffin-embedded and serially sectioned (5-mm sections). Then, these sections were deparaffinized, and endogenous peroxidase was inactivated by treatment with 3% (w/v) hydrogen peroxide. Citrate antigen retrieval solution (Beyotime, Shanghai, China) was used for antigen retrieval for 30 mins at 95–100 °C. Slide-mounted cell sections were incubated with an AMH antibody (ab103233, Abcam, Cambridge, UK) overnight at 4 °C and an HRP-conjugated secondary antibody for 1 h at room temperature. A GTVision III detection System/Mo&Rb kit (DAKO, K5007, Denmark) was used for subsequent visualization steps. Cell sections were stained with diaminobenzidine substrate, and nuclei were counterstained with hematoxylin. Light microscopy was used for image visualization and capture. Result quantified by image analysis software (ImageJ 1.51e, NIH Image).

### Immunoblotting

Immunoblotting was performed according to previously published protocols^40^. Mouse ovaries from each treatment group were homogenized in a lysis buffer (RIPA Lysis Buffer, Biosharp; protease inhibitor PMSF, Biosharp; and phosphatase inhibitors; Beyotime). After centrifugation (12,000 rpm, 10 min at 4 °C), the supernatants were collected and were held at 100 °C for 10 min. About 1 × 10^6^ KGN cells were washed twice in 4 °C PBS, then lysed by incubation with 300 µL radioimmunoprecipitation assay lysis buffer (89900, Thermo Fisher Scientific, Shanghai, China) for 10 min at room temperature and 10 min at 100 °C. Protein concentrations were determined with a BCA protein assay kit (A53226, Thermo Fisher Scientific, Shanghai, China). Equivalent amounts of protein were separated via sodium dodecyl sulfate-polyacrylamide gel electrophoresis, and transferred to polyvinylidene fluoride membranes (Immobilon, Millipore), following standard protocols. Membranes were incubated with a primary antibody, which was diluted with 5% evaporated milk, and then with an HRP-conjugated anti-rabbit secondary antibody (G-21234, Thermo Fischer Scientific, 1:5000 dilution). The primary antibodies used recognized AMH (ab103233, Abcam, Cambridge, UK, 1:100 dilution), glucose-regulated protein 78 (GRP78; 11587-1-AP, Proteintech, Wuhan, China, 1:1000 dilution), cyclic AMP-dependent transcription factor ATF4 (ATF4; BS6475, Bioworld, Nanjing, China, 1:1000 dilution), X-box-binding protein 1 (XBP1; BS70247, Bioworld, 1:1000 dilution), DNA damage-inducible transcript 3 protein (CHOP; BS1527, Bioworld, Nanjing, China, 1:1000 dilution), autophagy-related protein LC3B (LC3B; ab192890, Abcam, Shanghai, China, 1:2000 dilution), beclin-1 (ab207612, Abcam, Shanghai, China, 1:2000 dilution), P62 (P62/SQSTM1;18420-1-AP, Proteintech, 1:1000 dilution), mTOR (2983 T, Cell Signaling Technology, Danvers, MA, USA, 1:1000 dilution), Phospho-mTOR (p-mTOR, 5536 T, Cell Signaling Technology, 1:1000 dilution), p70 S6 Kinase (P70S6K, 2708 T, Cell Signaling Technology, 1:1000 dilution), and Phospho-p70 S6 Kinase (p-P70S6K, 9234 T, Cell Signaling Technology, 1:1000 dilution). Membranes were scanned with an Odyssey imaging system (Li-Cor, Lincoln, NE, USA), and proteins were quantified with Image Studio 1.1 software (Li-Cor). The grayscale of indicated protein was quantified by image analysis software (ImageJ 1.51e, RRID:SCR_003070). The experiment was repeated three times independently with similar results and all blots derive from the same experiment and were processed in parallel.

### Apoptosis assay

Apoptosis was detected with a FITC Annexin V Apoptosis Detection Kit I (556547) (BD, San Jose, CA, USA). KGN cells were treated with DMEM, CTX (750 µg/mL, 1000 µg/mL) or CTX (750 µg/mL, 1000 µg/mL) + GnRHa (100 µg/mL) for 36 h or 48 h. Then all cells, including floating and adherent cells, were collected by centrifugation, washed twice with PBS, and resuspended in 100 μL of binding buffer per 1 × 10^5^ cells. Following incubation with 5 μL of FITC Annexin V and 5 μL of PI solution in the dark at room temperature for 10 min, 400 μL of binding buffer was added, with gentle mixing. Cell signals were detected by flow cytometry with a FACS LSR II flow cytometer (Irawan, #30). All observations were reproduced at least three times in independent experiments.

### Reverse transcription-quantitative real-time PCR (RT-qPCR)

Total RNA was extracted using a fast kit purchased from Haigene Biotechnology (B0132, Haigene, Haerbin, China). Reverse transcription of total RNA was performed with the PrimeScript RT reagent kit (RR036A, Takara, Tokyo, Japan), and complementary DNA was then subjected to PCR with the Takara TB Green Premix Ex Taq kit (RR420A) in the ABI 7500 Real-Time PCR system (Applied Biosystems, Waltham, MA, USA). β-actin was used as a reference gene. DDCq method was used as analysis method. Real-time PCR experiments were repeated three times. Primers used for real-time PCR are listed in Supplementary Table 1.

### Confocal microscopy

KGN cells seeded onto glass coverslips in six-well plates at a density of 5 × 10^4^ were transfected with plasmids for expression of FLAG-tagged AMH and treated with CTX, GnRHa, or CTX and GnRHa. After 48 h, immunofluorescence staining assays were carried out, as previously described^39^, with primary antibodies for AMH (ab103233, Abcam, Shanghai, China) and GRP78 (AF5366, Affinity, Jiangsu, China), and secondary goat anti-rabbit antibodies conjugated to FITC or Alexa Fluor 594 (Proteintech). Images were visualized and captured with a TCS SP5 confocal microscope Leica (Shanghai, China).

### Transmission electron microscopy

Cell pellets were fixed in 2.5% glutaraldehyde at room temperature for 2 h and washed three times with 0.1 M PBS (10010023, Gibco, Shanghai, China). The pellets were post-fixed with 1% OsO_4_ for 2 h at room temperature and washed three additional times with 0.1 M PBS. The samples were dehydrated in a graded ethanol series followed by 100% acetone (650501, Sigma, Shanghai, China) and were placed in a 2:1 solution of acetone:epon 812 (45359, Sigma) overnight at room temperature. Finally, the samples were embedded at 60 °C for 48 h. Ultrathin sections (60–80 nm thick) were prepared using a Leica Ultracut UCT Ultramicrotome with a diamond knife, and stained with uranyl acetate (2%) for 15 min, followed by Reynold’s lead citrate staining for 15 min. Stained samples were viewed via transmission electron microscopy in an HT7700 microscope (HITACHI, Tokyo, Japan).

### Statistical analysis

Most experimental data are presented as the mean ± standard deviation. Two-tailed, unpaired Student’s *t* tests were used for the comparison of data between two groups. For data that were not normally distributed, the analysis used the Wilcoxon test. The chi-squared test was used for the analysis of numerical data. For data with more than two groups (such as the real-time PCR data), one-way analysis of variance (ANOVA) was used, whereas two-way ANOVA was used for comparison of follicle count data, followed by a Tukey test for multiple comparisons. A *P* value <0.05 was considered statistically significant. Statistical software SAS 9.4 was used for all data analysis.

### Reporting summary

Further information on research design is available in the Nature Research Reporting Summary linked to this article.

## Supplementary information

Supplementary Information

Reporting Summary

## Data Availability

The data that support the findings of this study are available from the corresponding author on reasonable request.
