# Supplementary material for: GnRHa protects the ovarian reserve by reducing endoplasmic reticulum stress during cyclophosphamide-based chemotherapy
Source: NPJ Breast Cancer. 2021 Oct 7;7:132. doi: 10.1038/s41523-021-00340-7 (PMC8497541; doi:10.1038/s41523-021-00340-7)
Supplement: Supplementary file 1 — Supplementary Information [file 41523_2021_340_MOESM1_ESM.pdf]

Supplementary Materials:

Supplementary Figure 1. Study design

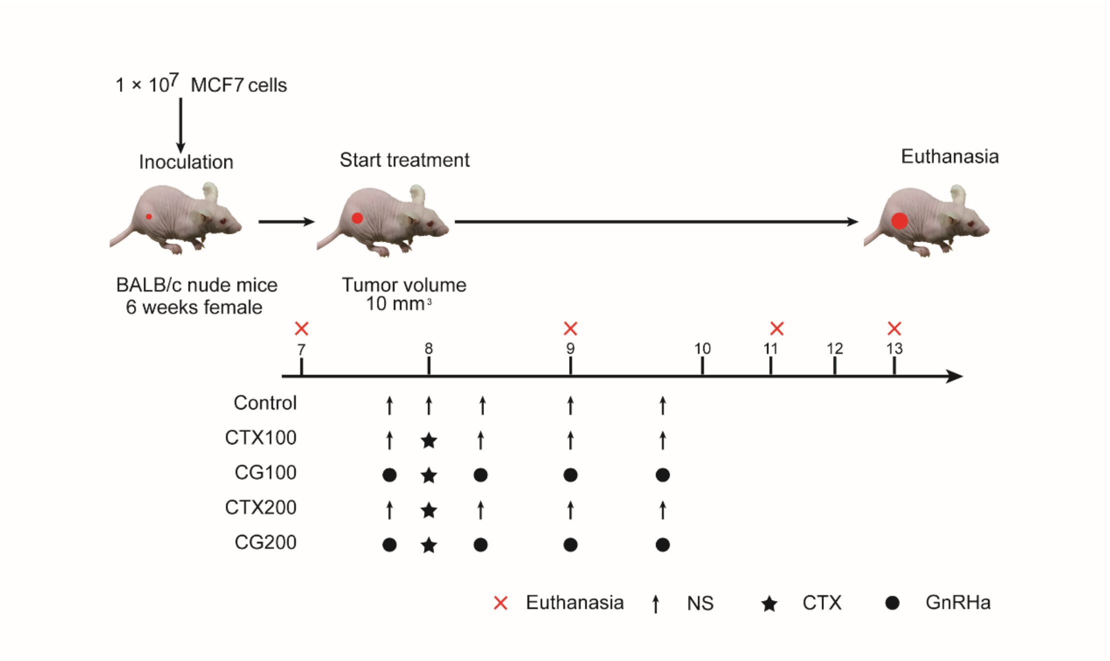

Timeline of experiments in tumor-bearing nude mice.

Supplementary Figure 2. Extended data for Figure 3b

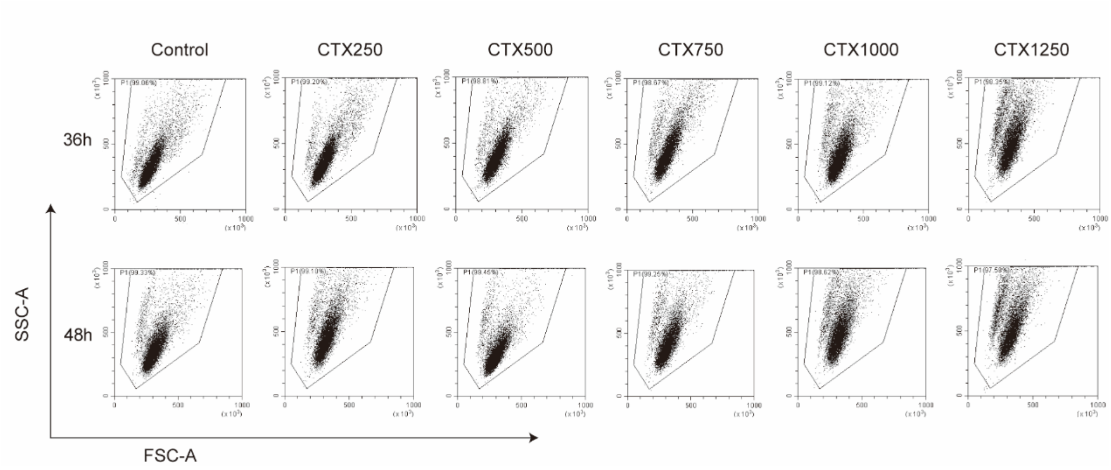

Supplementary figure of gating strategy for figure 3b.

Supplementary Table 1. PCR primer sequences referenced in the text.

AMH F	CCGAGACCTACCAGGCCAACAA
AMH R	GCATCTTCAGCAGCAGCACCA
GRP78 F	CTGCGTCGGCGTGTTCAAGA
GRP78 R	GGTTGGAGGTGAGCTGGTTCTT
ATF4 F	AACAAGACAGCAGCCACTAGGT
ATF4 R	CTTGCCTTGCGGACCTCTTCT
XBP1F	CCCTCCAGAACATCTCCCCAT
XBP1R	ACATGACTGGGTCCAAGTTGT
CHOP F	CTGCTTCTCTGGCTTGGCTGAC
CHOP R	CCGTTTCCTGGTTCTCCCTTGG
SEC12 F	CGCTTGAGTGCCTCCTTGCT
SEC12 R	CCTGCTGTTGATGTGCCTGGAA
SEC16 F	GTGCCACCAGCGTTCCTCATT
SEC16 R	TCACCTCGTCTCTTCCGTCACT
Actin F	CTACGTCGCCCTGGACTTCGAGC
Actin R	GATGGAGCCGCCGATCCACACGG
