# Supplementary material for: GnRHa protects the ovarian reserve by reducing endoplasmic reticulum stress during cyclophosphamide-based chemotherapy
Source: NPJ Breast Cancer. 2021 Oct 7;7:132. doi: 10.1038/s41523-021-00340-7 (PMC8497541; doi:10.1038/s41523-021-00340-7)
Supplement: Supplementary file 2 — Reporting Summary [file 41523_2021_340_MOESM2_ESM.pdf]

nature research

Corresponding author(s): Xiangyun Zong
Last updated by author(s): Sep 9, 2021

Reporting Summary

Nature Research wishes to improve the reproducibility of the work that we publish. This form provides structure for consistency and transparency in reporting. For further information on Nature Research policies, see our Editorial Policies and the Editorial Policy Checklist.

Statistics

For all statistical analyses, confirm that the following items are present in the figure legend, table legend, main text, or Methods section.

n/a	Confirmed	
☐	☒	The exact sample size (*n*) for each experimental group/condition, given as a discrete number and unit of measurement
☐	☒	A statement on whether measurements were taken from distinct samples or whether the same sample was measured repeatedly
☐	☒	The statistical test(s) used AND whether they are one- or two-sided<br>*Only common tests should be described solely by name; describe more complex techniques in the Methods section.*
☒	☐	A description of all covariates tested
☒	☐	A description of any assumptions or corrections, such as tests of normality and adjustment for multiple comparisons
☐	☒	A full description of the statistical parameters including central tendency (e.g. means) or other basic estimates (e.g. regression coefficient) AND variation (e.g. standard deviation) or associated estimates of uncertainty (e.g. confidence intervals)
☒	☐	For null hypothesis testing, the test statistic (e.g. *F*, *t*, *r*) with confidence intervals, effect sizes, degrees of freedom and *P* value noted<br>*Give P values as exact values whenever suitable.*
☒	☐	For Bayesian analysis, information on the choice of priors and Markov chain Monte Carlo settings
☒	☐	For hierarchical and complex designs, identification of the appropriate level for tests and full reporting of outcomes
☒	☐	Estimates of effect sizes (e.g. Cohen's *d*, Pearson's *r*), indicating how they were calculated

Our web collection on statistics for biologists contains articles on many of the points above.

Software and code

Policy information about availability of computer code

Data collection	Microsoft Excel 2019 MSO (1 6.0.13328.20334) 32 bit<br>Product ID: 00405-32271-13736-AAOEM<br>Session ID: F716C6DE-86B3-4544-9749-604550A15C81
Data analysis	Statistical software SAS 9.4 was used for all data analysis.

For manuscripts utilizing custom algorithms or software that are central to the research but not yet described in published literature, software must be made available to editors and reviewers. We strongly encourage code deposition in a community repository (e.g. GitHub). See the Nature Research guidelines for submitting code & software for further information.

Data

Policy information about availability of data

All manuscripts must include a data availability statement. This statement should provide the following information, where applicable:

- Accession codes, unique identifiers, or web links for publicly available datasets
- A list of figures that have associated raw data
- A description of any restrictions on data availability

The data that support the findings of this study are available from the corresponding author on reasonable request.

Field-specific reporting

Please select the one below that is the best fit for your research. If you are not sure, read the appropriate sections before making your selection.

☒ Life sciences ☐ Behavioural & social sciences ☐ Ecological, evolutionary & environmental sciences

For a reference copy of the document with all sections, see nature.com/documents/nr-reporting-summary-flat.pdf

Life sciences study design

All studies must disclose on these points even when the disclosure is negative.

Sample size

The animal experiment design follows the "3R" principle, including the replacement, reduction and refinement of experimental animals. Statistics require at least 6 available data in each group to be meaningful. According to statistics and previous studies, the sample size of animal experiment was determined

Data exclusions

No data were excluded from the analyses

Replication

We confirm that all results are repeatable

Randomization

Each mouse randomly assigned into one of five groups by simple random sampling.

Blinding

Investigators weren't blinded to mouse group in our research. Blinding was not relevant to our study.

Reporting for specific materials, systems and methods

We require information from authors about some types of materials, experimental systems and methods used in many studies. Here, indicate whether each material, system or method listed is relevant to your study. If you are not sure if a list item applies to your research, read the appropriate section before selecting a response.

Materials & experimental systems

n/a	Involved in the study	
☐	☒	Antibodies
☐	☒	Eukaryotic cell lines
☒	☐	Palaeontology and archaeology
☐	☒	Animals and other organisms
☒	☐	Human research participants
☒	☐	Clinical data
☒	☐	Dual use research of concern

Methods

n/a	Involved in the study	
☒	☐	ChIP-seq
☐	☒	Flow cytometry
☒	☐	MRI-based neuroimaging

Antibodies

Antibodies used

HRP-conjugated anti-rabbit secondary antibody (G-21234, Thermo Fischer Scientific); AMH (ab103233, Abcam, Cambridge, UK), glucose-regulated protein 78 (GRP78; 11587-1-AP, Proteintech, Wuhan, China), cyclic AMP-dependent transcription factor ATF-4 (ATF4; BS6475, Bioworld, Nanjing, China), X-box-binding protein 1 (XBP1; BS6857, Bioworld), DNA damage-inducible transcript 3 protein (CHOP; BS1527, Bioworld, Nanjing, China), autophagy-related protein LC3B (LC3B; ab192890, Abcam, Shanghai, China), beclin-1 (ab207612, Abcam, Shanghai, China), P62 (P62/SQSTM1;18420-1-AP, Proteintech), mTOR (2983T, Cell Signaling Technology, Danvers, Massachusetts, USA), Phospho-mTOR (p-mTOR, 5536T, Cell Signaling Technology), p70 S6 Kinase(P70S6K, 2708T, Cell Signaling Technology), and Phospho-p70 S6 Kinase(p-P70S6K, 9234T, Cell Signaling Technology).

Validation

See the antibody instruction sheet of manufacturer's website for details.

Eukaryotic cell lines

Policy information about cell lines

Cell line source(s)

The KGN steroidogenic human ovarian granulosa-like tumor cell line was obtained from Guandao Bioengineering (Shanghai, China). The MCF7 human breast cancer cell line was obtained from the American Type Culture Collection.

Authentication

Each cell line was authenticated by Multiplex - PCR (STR) DNA profiling.

Mycoplasma contamination

We declared that the cell lines were not tested for mycoplasma contamination.

Commonly misidentified lines (See ICLAC register)	NA

Animals and other organisms

Policy information about studies involving animals; ARRIVE guidelines recommended for reporting animal research

Laboratory animals	Balb/c-nu mice (6 weeks old, 18–20 g, female, obtained from Shanghai SLAC Laboratory Animal Co., Shanghai, China)
Wild animals	NA
Field-collected samples	NA
Ethics oversight	All animal procedures were approved by the Animal Ethics Committee at Shanghai Jiao Tong University Affiliated Shanghai Sixth People's Hospital and were carried out in accordance with the 'Australian Code of Practice for the Care and Use of Animals for Scientific Purposes'

Note that full information on the approval of the study protocol must also be provided in the manuscript.

Flow Cytometry

Plots

Confirm that:

- [x] The axis labels state the marker and fluorochrome used (e.g. CD4-FITC).
- [x] The axis scales are clearly visible. Include numbers along axes only for bottom left plot of group (a 'group' is an analysis of identical markers).
- [x] All plots are contour plots with outliers or pseudocolor plots.
- [x] A numerical value for number of cells or percentage (with statistics) is provided.

Methodology

Sample preparation	KGN cells were treated with DMEM, CTX (750 µg/mL, 1000 µg/mL) or CTX (750 µg/mL, 1000 µg/mL) + GnRHa (100 µg/mL) for 36 h or 48 h. Then all cells, including floating and adherent cells, were collected by centrifugation, washed twice with PBS, and resuspended in 100 µL of binding buffer per 1 × 105 cells. Following incubation with 5 µL of FITC Annexin V and 5 µL of PI solution in the dark at room temperature for 10 min, 400 µL of binding buffer was added, with gentle mixing.
Instrument	CytoFLEX LX and BECKMAN COULTER
Software	Flow Cytometry Software: CytExpert 2.3
Cell population abundance	Flow cytometry was used to detect the apoptosis of KGN cells treated with different concentrations of CTX.We mainly observed the ratio of apoptotic cells to all cells.
Gating strategy	The operating software of the flow cytometer was used to set the X axis with FSC and Y axis with SSC, and the FSC/SSC gain and FSC threshold were adjusted to make all the cells appear. The target cell group was selected by the gate tool circle, which accounted for more than 80% of all the particles and was identified as the target cell group. The "negative" refers to the blank cell group without staining.

- [x] Tick this box to confirm that a figure exemplifying the gating strategy is provided in the Supplementary Information.
